# Social inclusion among young adults with mental illness and complex needs in Flexible Assertive Community Treatment: A qualitative study on team staff’s understanding and experiences

**DOI:** 10.1371/journal.pone.0341358

**Published:** 2026-01-23

**Authors:** Silje Nord-Baade, Ottar Ness, Michael Rowe, Camilla Bergsve Jensen, Anne Landheim

**Affiliations:** 1 Norwegian National Advisory Unit on Concurrent Substance Abuse and Mental Health Disorders, Innlandet Hospital Trust, Hamar, Norway; 2 University of Inland Norway, Elverum, Norway; 3 Norwegian University of Science and Technology, Trondheim, Norway; 4 School of Medicine, Yale University, New Haven, Connecticut, United States of America; University of the West Indies at Saint Augustine, TRINIDAD AND TOBAGO

## Abstract

**Background:**

Social inclusion is increasingly highlighted through policies, practices, research, and broader societal awareness. Young adults with mental illness and complex needs are among the most marginalized in society and face significant challenges. Public health and welfare services play a critical role in either facilitating or hindering social inclusion through adequate or inadequate service provision. The services are often critiqued for treating individuals in isolation from their social contexts.

**Methods:**

In this qualitative study semi-structural individual interviews and thematic analysis were used to explore: How do service providers in FACT teams understand and promote social inclusion among young adults with mental illness and complex needs?.

**Results:**

The results showed that staff employed efforts to promote social inclusion and acknowledged this as important. However, they had limited conceptual understanding and revealed a lack of systematic promotion in the teams. Several barriers for social inclusion were evident in the material, from individual and structural to societal.

**Conclusions:**

There is a need to develop and operationalize the concept to enable systematic and holistic approaches to promote social inclusion, moving beyond employing single interventions. Policies must be developed, and training and education provided to create context sensitive mental health services.

## Introduction

Despite being known as a well-functioning welfare state with high scores on social capital measures relative to many other countries [1], social exclusion among young adults represents a major societal concern in Norway, as with many other countries. Social exclusion as both a cause and result of mental health and substance use problems has increasingly been highlighted through policies, practices, research, and broader societal awareness [2,3]. The relationship between social inclusion and enhanced quality of life, physical, and mental wellbeing is well documented, as is the correlation between social exclusion and higher rates of mortality, morbidity, poverty, poor physical and mental health, and reduced quality of life [2–4].

The concept of social inclusion is debated and defined in numerous ways [5–7]. Work, education, housing, neighborhood, social activities, and social support are most frequently mentioned across definitions [5]. Still, ambiguity constitutes a challenge related to creating a common understanding. In general terms, social inclusion can be defined as a process that improves social participation across different marginalized groups “through enhanced opportunities, access to resources, voice and respect for rights” (3, p. 20). Social exclusion, by contrast, can be defined as “a state in which individuals are unable to participate fully in economic, social, political and cultural life, as well as the process leading to and sustaining such a state” (3, p. 18). These definitions, although relevant, are broad and can be interpreted in many ways and at different levels. Social exclusion and promotion of inclusion among young adults, are often understood in terms of the NEET (Not Employed, Education or Training) concept [8]. Thus, acquiring work, being enrolled in education or training are central to promoting social inclusion in health and welfare services. To allow for an exploratory and context-sensitive approach to how the concept is interpreted and enacted in practice, the present study will not confine the understanding of social inclusion to a single definition or theoretical framework.

The 18–29 age range constitutes a crucial developmental period. During this time, young adults normally strive for autonomy, develop emotional stability, establish their identity, form adult relationships, and set a career path. Additionally, they explore new interests and learn to manage practical aspects of life. Thus, this developmental phase is particularly important for laying the foundation for a healthy adult life [9]. Success in negotiating this natural process is challenging for many, and even more so for those with mental illness and complex needs. Young adults in this group frequently encounter challenges related to stigma, interpersonal connections and social reciprocity, vocational engagement, autonomy, unhealthy identity development, independent housing and stability [10–13]. Thus, this group is among the most marginalized in society [2,3,14], underlining the importance of social inclusion efforts.

There are several identified barriers to and facilitators of social inclusion. Young adults with mental illness and complex needs experience subjective barriers to social inclusion in addition to challenges related to such concrete elements as income, housing and employment, with identity and sense of ‘not mattering‘ as important subjective barriers [13,15,16]. Public health and welfare services play a crucial role in either facilitating or presenting barriers to social inclusion, often criticized for treating individuals in isolation from their social contexts and not prioritizing interventions aimed at social inclusion [17–23].

Social inclusion process of young adults with mental illness and complex needs is understudied [24]. Research points to inadequate attention to social and clinical factors in promoting social inclusion among this group [25]. Little is known about how mental health providers understand promotion of social inclusion, and research on holistic social inclusion interventions and practice-oriented frameworks is scarce [26]. Interventions that focus on accommodation, supported employment, and family psychoeducation foster social inclusion, although the latter is less frequently employed than the first two. In addition, there is a lack of research on social skills interventions. However, there is growing evidence on the benefits of peer-supported interventions [26]. Promotion of social inclusion among this group is challenging and requires a holistic approach with attention to both the person’s needs and the service context in which they are addressed.

Implementation of Flexible Assertive Community Treatment (FACT) represents a positive development in Norwegian services [27,28]. FACT is a service model that provides integrated care for people with severe mental health and/or substance use problems who have complex needs [29]. The teams provide person-centered and long term care through a flexible approach, enabling the staff to scale the intensity of support based on individual needs. The staff typically consists of psychiatrists, nurses, social workers, psychologists, vocational specialists, and peer support workers. They operate on a shared caseload model, meaning that all team members collaborate to enhance the individual’s recovery process. Social inclusion is an integral part of the model, with service provision in local communities and simultaneous attention to employment, education, and meaningful activities. The model also underlines the importance of a community-wide approach including people’s families, social networks and neighborhoods. Current FACT teams promote social inclusion to a degree [e.g., 30], but is often seen and reported in relation to outcome measures such as recovery processes and service efficiency, or based on user experiences [27,28,31,32].

The aim of this study is to provide practice-oriented knowledge to guide future research and practical implications to enhance social inclusion. Thus, our research question is*: How do service providers in FACT teams understand and promote social inclusion among young adults with mental illness and complex needs?*

## Materials and methods

### Design and paradigm

The study employed a qualitative, exploratory, and participatory design, drawing on social constructionism theory [33], which views knowledge as co-created, integrating the factors and dynamics of both personal and social contexts. A participatory design was chosen to ensure the study’s relevance and validity, with attention to both lived experience and academic knowledge [34]. The relevance of the study was discussed with several people with lived experience, including two young adults who were members of a national advisory board on the implementation of Youth FACT in Norway. These discussions, which revealed that few had been asked about their needs and wishes for social inclusion, confirmed the necessity of this study. Additionally, a peer researcher has been involved in the research process from the planning stage to analysis and dissemination of results.

### Research context

The study is part of a larger project involving promotion of social inclusion among young adults and staff in FACT, recruited from three teams in Eastern parts of Norway. All teams provided services to people in both urban and more rural areas. Two teams had been operating since 2020 and one since 2021. These teams integrated expertise from the specialist and municipal health care services and peer workers. All teams had received basic training on the FACT model and participated in professional FACT networks that provided opportunities for continued professional development.

### Recruitment and participants

To recruit participants, we approached team leaders in three teams. After receiving information on the aim of the study, the team leader agreed to inform the team staff about the study, encouraging them to participate. We do not know how the staff was informed. All team members, regardless of profession or role within the teams, were eligible for participation to gain experiences from different perspectives within the teams. Eligibility required experience in working with young adults with mental illness and complex problems in a FACT setting. Those interested in participating received written information about the study and their rights, and a form of consent. Recruitment was challenging, leading to six participants. The first author contacted and scheduled interviews with people who consented to participate.

Six participants (four women and two men) participated in the study. All but one had extensive experience working with young adults with mental illness and complex problems. The participants had basic education in nursing, some with specialized training in mental health work, clinical psychology and in child and youth care work. All participants disclosed having received none to limited education about social inclusion, both in basic education and afterward.

We used the pseudonyms Emma, Helen, Robert, David, Laura and Alice for the participants.

### Data collection

Data was collected from May to September 2024 through individual semi-structured interviews. This approach was selected to maximize the potential for gathering rich descriptions and a deep understanding of participants’ experiences [35]. Individual interviews were chosen to ensure credible data material, as participants were able to express their views anonymously. An interview guide was developed by the first author followed by review and discussion with other authors. This collaborative process reduced the need for pilot testing. As a result, pilot testing was not conducted.

The first author conducted the interviews. The participants chose the interview location to ensure a comfortable and safe setting for sharing their experiences. The first author also reassured them that all information would be treated confidentially, especially if they disclosed critical issues or negative experiences. Four participants were interviewed in person at their FACT teams’ headquarters and two by online video interview. The interviews took form as conversations, emphasizing the value of the participants’ experiences.

The aim was to explore the participants’ understanding of social inclusion as a concept and their views and experiences with promoting it for young adults in their teams. They were also asked about collective understanding and views on joint efforts as a team, and about perceived barriers and room for improvements. In addition, they were briefly presented with some main results from previous studies addressing the experiences of social inclusion among young adults in FACT teams [13,36], and asked to reflect on these.

The interviews were experienced as honest and open conversations. Follow-up questions were asked to clarify or deepen responses, and participants were invited to provide specific examples to illustrate their experiences. All were given the chance to provide additional statements after the interview, of which none did. The length of interviews was 50–70 minutes, with an average duration of 57 minutes, and provided rich material with acceptable information power [37]. The interviews were recorded and transcribed verbatim by the fourth author.

### Analysis

We employed and followed the six phases of reflexive thematic analysis [38] as described below. This enabled us to explore the descriptions and meanings of social inclusion through emphasizing the voices of the participants, and examining their comments in relation to individual, social and structural factors reflected in their views [33,39]. A team approach was employed.

The first and fourth authors, the latter a peer researcher, thoroughly reviewed the data and discussed their initial impressions to become familiarized with the dataset (phase 1). The peer researcher initially shared her impressions and thoughts to minimize the influence of the non-peer researcher. Both authors’ initial impressions and reflections were highly similar. Following this, the first author coded the interviews (phase 2), introducing initial thoughts to all contributing authors. After this, initial themes were generated (phase 3), then further developed and reviewed with contributing authors (phase 4), and finally refined, defined and named (phase 5). Throughout these stages, the authors endeavored to exercise critical awareness to avoid biases in interpretation of the data. There were discussions with all authors during all phases of the analysis [40], and the material was read several times to re-examine initial findings. There were some movements back and forth between the different phases before writing up the results (phase 6). Three main themes were developed: [1] “It has something to do with…”, [2] “We really try”, and [3] “We could do more” with a total of six sub-themes across the three main themes.

### Ethical considerations

The study was approved by the local data protection officer at Innlandet Hospital Trust (ID 22587532), following recommendations by the Regional Committee for Medical and Health Research Ethics in Norway (482463). The participants signed a consent form with information stating that the interviews were confidential, that statements would have no impact on their work life, and that they could withdraw from the study at any point. This information was repeated orally before the interviews started. Participants were told that their contributions and experiences were important, and they were given the chance to ask questions or voice concerns before participating in the study. None did. At the end of the interview, the researcher asked about the participant’s experience of the interview to ensure that no harm was inflicted. Participants rated their experiences as positive.

## Results

Three main themes were developed: [1] “It has something to do with…”, [2] “We really try”, and [3] “We could do more” with a total of six sub-themes. All staff agreed on the importance of promoting social inclusion and employed various means to do so. However, in practice, they experienced difficulties in operationalizing the concept, barriers for the young adults themselves, limitations in how the teams operated and the workload, in addition to overarching social and structural challenges.

### “It has something to do with…”

The first main theme reveals the team staff’s understanding and awareness of social inclusion in general. It encompasses two subthemes: Defining social inclusion and level of awareness, and Collective understanding and team awareness.

#### Defining social inclusion and level of awareness.

Participants were asked to define social inclusion and highlight important elements of it. All said they found it challenging to provide a clear definition. Some, such as Helen, primarily provided overarching statements related to belonging or being a part of society:

“For me, it sounds like being part of society. Being able to contribute or be part of that society, not being completely isolated in a way. That’s what I think.”

After further exploration, most participants highlighted education, work, participating in meaningful activities, and interacting with friends and family as core elements of social inclusion. Statements also referenced social inclusion as a human right, and on social inclusion as not merely an issue involving individual clients but as including societal responsibilities and barriers:

“[Social inclusion is] to feel that one is part of society. That one gets to participate on the same terms as everyone else. Not to be someone who observes, but someone who is included. I think about that in all contexts. That is, to feel that one is allowed to have a job. One can study if one wants. That there are opportunities for me to meet a partner, friends, and engage in leisure activities. There are arenas where I am welcome, where I feel that I can take my place and exist. […] There should be opportunities, even if you’re not in a good situation. That should not be a limitation.” (Alice)

In addition to defining social inclusion, the participants were asked about their level of awareness and the attention given to social inclusion in their daily interaction with the young adults. All acknowledged both the importance of social inclusion and the lack of their and their teams’ specific attention to it. Rather, social inclusion was present in their thinking, but without specific knowledge of the concept in regard to treatment and follow up. Thus, all participants recognized the importance of social inclusion, but their understanding of it appeared more in line with professional experience and common sense related to social needs, rather than practice-oriented theoretical knowledge.

#### Collective understanding and team awareness.

All participants said their teams addressed social inclusion, but none remembered discussing or reaching consensus on a collective understanding of what social inclusion is. Participants were unsure and doubtful as to fellow team members having a common perception of social inclusion, Emma said:

“I don’t really dare to say exactly how they (fellow team members] think about it. This could mean that we might not have a very common understanding of it. Or awareness. We don’t talk about it from any specific model other than recovery.”

Others experienced having such conversations in the teams but as related to engaging young adults in activities or efforts to connect them with the teams’ IPS (Individual Placement and Support) worker. One participant spoke of the 5 R’s of Citizenship [41] being mentioned briefly in a team discussion, but did not recall the context of or discussion of the concept. In this and other comments, participants’ sense of how social inclusion was promoted and addressed was dependent on individual stat, with no systematic approach by the team, suggesting potential variations in means offered to promote social inclusion.

In sum, participants perceived the teams as having a general awareness of the importance of social inclusion but not a systematic and holistic approach to it in their teams.

### “We really try”

In addition to exploring how the staff defined social inclusion, participants were asked about how they understood the young adult’s social context and needs and how they worked to promote their social inclusion.

#### Understanding young adults’ social context and needs.

Some participants were uncertain as to whether the young adults recognized themselves as socially excluded or saw it as problematic. It was noted that young adults spoke at times of having social needs met through social networks, albeit not always with positive influences. Others noted that some young adults longed for closer contact with family and friends, and recognition as being ‘part of something,’ but were not given a second chance to do so.

Some participants said their clients lacked awareness or interest in broader social opportunities, keeping a distance from social activities, education, and work due to not seeing themselves as socially marginalized. Several perceived a general lack of motivation to engage as a main problem. Others highlighted a sense of hopelessness among some young adults, making it difficult to identify their needs and engage them in efforts to address exclusion. Alice said:

“They must come to the realization that *this is a problem I have [needing treatment and being excluded], that I want help with*. I don’t really think they always realize that they have a problem, and still, everyone else does. And at the same time, it’s like they say *it’s already over for me, fuck, it’s shit anyway*.”

When comparing promotion of social inclusion among younger versus older patients, some participants said they didn’t see any differences between the two groups or that they hadn’t thought about it. Some had clearer opinions, experiencing older patients as more difficult to motivate for change because they were set in their ways. Others had the opposite experience. This was partly explained by what they saw as a lack of interest in society. Others spoke of difficulties in reaching young adults and engaging them in both treatment and pursuit of social inclusion. This was related to them not having experienced major consequences, such as somatic health issues where change was connected to a matter of life or death. Robert said it like this:

“They are difficult to get hold of. I think they hide much more inside, they are much more anxiety ridden. They use substances more quietly. It’s harder to get them to participate in activities or work, than those over 30 in a way. […] I think maybe those who are older have gained a bit more experience and see it more appropriate to look for a change in their life to be able to do something useful. I think they pay more attention to what’s going on in the society in general and begin to see what they are missing out on. […] They become more interested in hearing about the possibilities and want to participate in it. […] Simply more maturity, and that they have been in the treatment systems longer and maybe found out they need to use the services in a different way.”

Summing it up, the participants expressed uncertainty about whether all marginalized young adults recognized their own social exclusion and saw it as problematic. Further, staff described their clients’ lack of motivation and interest in society as a factor in their social inclusion.

#### Promoting social inclusion in practice.

To explore how the team staff worked to promote social inclusion in practice, they were asked to provide examples from their work. All said they were trying to engage and help their clients make links to society.

Most staff spoke about working towards inclusion for their clients through supporting their participation in social activities or by obtaining work. Other factors such as income, housing, working on relationships and network were less frequently highlighted as a means to promote social inclusion, although they routinely collected information on available activities for their clients that were appropriate for their age group and their individual needs and interests. Some, however, experienced difficulty in finding such activities for young adults. Furthermore, engaging clients in meaningful activities was not always connected to social inclusion as an overarching goal. Helen said:

“It’s maybe not so much to get him [a patient] into society, it’s more so that he has something else to think about.”

The second primary means of supporting social inclusion appeared to be focusing on paid work or education for clients. However, after evaluating experiences with Individual Placement and Support in the teams, one team was in a process of redefining the role of their job specialist. They saw the need to create more space to engage patients in activities, both for the patients who didn’t seek work or education, and for those where it proved too difficult given their current situation. Another team had come to the same conclusions and had already included the activity focus in the job specialist role. Emma spoke of the risk of simultaneously reducing focus on work and inherently reducing expectations:

“Maybe it works better in this target group. I feel that I perhaps could promote social inclusion better that way. But I’m unsure of it, because I know that forcing yourself to think about work when it seems completely hopeless and impossible, sometimes it works when you least expect it.”

Staff did not speak about exploring their clients’ subjective experiences with them, such as that of identity or feeling valuable, in relation barriers to or facilitators of social inclusion. They did speak of encouraging their clients to examine their strengths and resources to help them address what they saw as their negative mindsets. Some spoke of having conversations with them regarding confidence and social skills in relation to suitable workplace behavior. Clearing up misunderstandings and challenging self-stigma was also mentioned. Laura experienced it as hard work: “How can I convince you that you matter to someone? It’s quite heavy work.” David spoke of this, pointing to differences in how these perspectives were addressed within the teams:

“Those aren’t conversations I usually get into, for the most. But I know that, from what I’ve heard, there’s a focus on self-esteem and value and such.”

Team staff primarily worked to promote social inclusion by engaging patients in meaningful activities and through obtaining work or education. Addressing psychological barriers was not initially highlighted but recognized as important when interviewees were asked directly about this.

### “We could do more”

#### Targeted areas of improvement.

The staff were asked to reflect on areas of improvement after sharing their experiences on how they promoted social inclusion in practice. All participants highlighted the use of meaningful activities as a means to promote social inclusion. Still, some experienced room for improvement via greater variety in what they offered. They also stated that they could do more to engage their clients in social activities. Robert spoke of the need for a more salutogenic and person-centered approach when offering activities, also mentioning a need to enhance work and cooperation with patients’ families:

“I think we need to explore much more to find their interests, dig a little. And have a bit more cooperation with family where we have the opportunity. Who is he [patient] really? We know him now, but who was he when he was younger? What did he like to do and what were his interests? What are his strengths? […] That’s where you can kind of look for the healthy aspects.”

Linked to exploring patients’ interests and strengths, participants mentioned improving how they communicated with the clients regarding social inclusion. Some noted that providers sometimes avoid pushing too hard in fear of doing harm to or provoking relapses among their clients. Others spoke of the need to better explain the rationale to their clients for employing social inclusion means, like Alice:

“If you don’t understand why, see the reason, see where you’re headed, if you don’t have a clear understanding of what the FACT team is doing for you and why you should keep going. As with the healthcare system in general, but if you don’t understand it you leave.”

Staff spoke of needing to create a common understanding in the teams and working more systematically. As some pointed out, unless it was put on the agenda, it would drown in everything else. One suggestion was to include goals for social inclusion in the patients’ treatment plans.

In summary, the participants reflected on areas of improvement in promoting social inclusion, emphasizing the need for a greater variety of meaningful activities and a more person-centered approach. They highlighted the importance of understanding patients’ interests and strengths, improving communication about social inclusion, and involving patients’ families. Additionally, participants noted the necessity of addressing social inclusion systematically within the team to ensure it remained a priority.

#### Perceived barriers.

All participants stated that they tried to promote social inclusion but experienced several barriers in addition to areas of improvement in their teams.

One oft-mentioned barrier involved challenging motivational work related to clients with complex problems and low levels of functioning. Additionally, frequently client crises and urgent basic needs were time consuming activities that constricted the room for doing planned work. Robert said: “I don’t think it’s due to unwillingness, it just gets so hectic sometimes.”

Another identified barrier was what interviewees described as the individualistic and medical focus of services. As Alice said, “I’m really trying, but it’s easy to fall into this disease-focused mindset.” This was perceived less problematic within FACT teams but as still having room for development. Another barrier related to the teams was their clients’ lack of economic means to engage in activities. Further, one participant mentioned that the teams were not operating in the evening, thus excluding all activities taking place after closing time.

The participants also mentioned barriers in the community. Some spoke of economic hardship in the municipalities, leading to closure of meeting places where clients could practice their social skills and gain positive experiences. Others mentioned prejudice and stigma, with one example being a local gym that had closed the door for FACT patients out of fear of antisocial behavior. Small communities were also considered to be a barrier both due to limited amounts of available activities and because of the negative reputation some patients had required. Another barrier was connected to employers. Emma said:

“I’ve been dreaming of a much more generous work life, really. That it’s safer to try someone who doesn’t have a great resume or a lot of experience, that has struggled to function in everyday life. […] I wish they [employers] could see it as a societal responsibility to include those who have fallen on the outside. […] And that the requirements weren’t so high all the time with expectations of full-time positions, that there could be a bit more room for variation.”

Summing up, the participants experienced barriers across different levels, spanning from the individual to societal, including the health and welfare services in general and the one they provided.

## Discussion

The aim of this study was to provide practical knowledge to guide future research and practice to enhance social inclusion, through exploring the following research question: *How do service providers in FACT teams understand and promote social inclusion among young adults with mental illness and complex needs?*

The De Forente Nasjoner [3] definitions on social inclusion and exclusion involve all levels, from individual to community to society. Our results show that FACT teams primarily focus on promoting social inclusion at the individual level, in accordance with their mandate [29], albeit touching upon community levels through cooperation with other stakeholders related to activities, work or education. Furthermore, the teams are guided by national policies and guidelines for the mental health services in which social inclusion is described as important, but in non-specific terms related to engagement in work, activities or education [42].

Despite recognizing the importance of social inclusion, our results show that the staff faced significant barriers within their clients, in their teams, and in the broader community. They reported employing efforts regarding work, education, and social activities in line with current knowledge of social inclusion interventions [26]. However, this study’s finding reveals the lack of an overarching understanding going beyond the commonsense aspect of participating in one’s community. This gap appears to hinder clear communication on social inclusion both within the teams and with young adults, resulting in barriers to promoting social inclusion systematically. The results of the present study underline the complex interplay between individual efforts, communities, and societal conditions regarding social inclusion, highlighting the need for increased knowledge and systematic approaches. The criticism that health and welfare services are too individualistically oriented and that they neglect social context in treatment [17–23], was partly evident in the staff’s experiences. Still, it should be noted that FACT teams represent an improvement in this area compared to ordinary services [30].

### The concept of social inclusion and who gets to define it

Our findings also raise questions about the practical usefulness of the concept of social inclusion in its current form, as its full potential is not being realized. Yet while the concept may be open to critique, it recognizes the inherent worth of every individual in our communities and the imperative to inequality and marginalization [3]. As such, social inclusion is a concept that deserves attention and continuous improvement.

Staff struggled to provide a working definition or understanding of social inclusion and its core facilitators going beyond work, education and activities. That said, these means are important facilitators of social inclusion in the FACT handbook [29]. Still, social inclusion is recognized as a complex problem [26] that requires more than isolated good initiatives to be effectively addressed. All staff interviewed thought their team members were aware of the importance of social inclusion, albeit with variation in the attention they gave to it. Interviewees could not recall team discussions aimed at reaching a common understanding of how they could systematically work as an interdisciplinary team to promote their patients’ social inclusion. This is an important finding, given that the FACT model clearly emphasizes social inclusion as a main goal through interdisciplinary efforts [29].

The staff reported a lack of formal education on the subject, both in their basic training and ongoing professional development, and further noted that they largely lacked systematic approaches in the teams. This supports the critique of mental health services not sufficiently including the social context in treatment (e.g., 22). However, all staff acknowledged the importance of social inclusion and employed efforts to promote it, while also calling for more practice-oriented knowledge.

One reason for the difficulties in working systematically to promote social inclusion may be the conceptual challenges of social inclusion and who gets to define it. Regarding conceptual challenges, staff observed young adults who often did not recognize their social exclusion or perceive it as problematic, suggesting differing views on social inclusion and its relevance. The staff acknowledged a need to further explore their clients’ individual needs, interests and resources and address social inclusion explicitly. Lack of clear definitions or frameworks for social inclusion is part of the problem, but the means for promoting and communicating about it follows close behind. This dilemma is in line with previous research showing that young adults in FACT teams have limited experience of service providers addressing social inclusion [13,36]. This suggests that young adults had limited influence in defining what social inclusion means to them and in determining the methods to promote it. Consequently, some individuals’ needs may be overlooked or inadequately addressed.

Additionally, there is a lack of integration of objective perspectives of social inclusion such as obtaining work or income, with subjective perspectives such as a sense of identity or perceptions of their social surroundings. Such aspects might be addressed in relation to the young adults’ mental illness, especially considering the young adults’ developmental phase. However, they should also be addressed in relation to social inclusion as these young adults often have experiences of relational traumas challenging how they interact with others, feeling labelled as worthless to society, of stigma and internalized stigma, in addition to experiencing limitations because of mental illness and complex needs [13,15,16,43,44]. Practitioners, researchers and policymakers must understand the dynamics of social inclusion for young adults with mental illness, including what society has to offer them and what they have to offer society. It is difficult to see how social inclusion can be promoted without touching upon the more subjective and existential aspects of what it means to be a human being in a modern society, nonetheless a human being challenged by complex needs.

Discussion and debate on social inclusion reveal significant challenges due to the vagueness of the term and the lack of a universally accepted definition. Social inclusion is often described in broad terms, without a clear consensus on its core components [5–7]. Such ambiguities negatively affect the creation of coherent policies, as policymakers struggle to operationalize the concepts, such as social inclusion, that they are charged with addressing, thus risking fragmentation and lack of coordination of effort [45,46]. As our results show, unclear concepts may lead to disparities in what support is offered. This study, in tandem with debates and criticism of the concept itself, raises important questions: Who gets to define social inclusion? Does the definition fit the perceived needs among the marginalized? Can strategies be devised and promoted for its successful implementation among service providers and authorities?

Further, as the definition remains unclear, difficulties in measuring social inclusion follow [47], making the effectiveness of interventions difficult to evaluate. Having accurate measures may help identify personal experiences of exclusion, enabling tailored support and resources to address specific needs. It may also empower people’s mental health literacy and clarify for them the meaning of their own inclusion (and exclusion) [48].

### Acknowledging the barriers

The staff spoke of individual barriers which primarily involved psychological and emotional challenges. These barriers not only affect individuals’ self-perception and mental well-being; they also impeded their participation in societal activities, education, and employment [13,36,44,49,50]. Participants in the study highlighted issues such as a perceived lack of motivation, feelings of hopelessness, and internalized stigma. As mentioned, one staff member described how some young adults viewed their situations as hopeless. Others noted that many clients did not recognize their social exclusion or see it as problematic, which made it challenging to engage them in activities aimed at inclusion. Additionally, some clients avoided broader opportunities for education, work, or social engagement because they did not see themselves as socially marginalized, reflecting a disconnect between their self-perception and societal expectations. These barriers underline the importance of interventions that build self-esteem, challenge negative self-concepts, and foster hope and agency.

Even if our results had shown that there was a common understanding of what social inclusion is and how it can be promoted, the goal of social inclusion relies not only on the excluded individual and the providers who assist them. It also relies on social and structural conditions in communities [50–52]. In the present study, the structural barriers involved systemic limitations within the FACT service framework. Participants frequently cited limited team resources and insufficient funding at the municipal level as significant obstacles. For instance, the lack of evening activities or limited access to appropriate social arenas in the local community was mentioned as a recurring issue. Additionally, the staff’s availability was limited to daytime hours, restricting their ability to support young adults who wished to engage in evening activities.

One staff member noted that the team’s focus was often diverted by frequent crises and urgent basic needs, leaving little room for planned, long-term inclusion efforts. Additionally, economic constraints in municipalities led to the closure of community meeting places, which further restricted opportunities for young adults to practice social skills and engage in meaningful activities. These structural challenges highlight the need for increased funding, expanded team operating hours, and better resource allocation to enable more inclusive and flexible services.

Societal barriers arise from broader social dynamics, such as stigma, prejudice, and exclusion within local communities. Participants described how negative attitudes from employers and community members limited opportunities for young adults to engage socially or professionally. For example, a staff member recounted how a local gym refused access to FACT clients due to concerns about antisocial behavior, effectively excluding them from an important social arena. Additionally, staff noted that some employers were reluctant to hire individuals with limited work experience or mental health challenges. These societal barriers not only impact young adults’ ability to participate in education, employment, and community activities but also reinforce feelings of marginalization and worthlessness. Addressing these barriers requires coordinated efforts to reduce stigma, raise awareness, and foster inclusive attitudes across communities. Countering such barriers requires efforts beyond the scope of FACT teams, thus pointing to the importance of helping young adults to handle social exclusion and supporting their capacity for resilience.

While these barriers can be categorized as individual, structural, or societal, they are deeply interconnected and often reinforce one another. The staff recognized that promoting social inclusion requires efforts that extend beyond the individual and FACT teams, relying heavily on broader social and structural conditions within communities. Still, FACT has been found to form bridges between different services [53] and might have an unused potential in forming bridges with other parts of the community as well. As others point out, promoting social inclusion is a complex matter and needs to involve multiple stakeholders [26].

### Implications and recommendations

#### Developing the concept of social inclusion, a framework and measures.

Our findings point to the need for further development of the concept of social inclusion, and operationalization in the context of a person-centered framework fitted to the needs of young adults. Such a framework must include both subjective and objective perspectives enabling all barriers to be addressed and resources to be discovered. This could be done through adapting knowledge from different sources such as research, lived experience, and professional expertise within a participatory co-creation approach drawing on the assets and experiences from different stakeholders in the community. Some studies have placed the lived experience of clients at the center in research aimed at understanding core elements of social inclusion [e.g., 54,55], providing valuable insights. How these findings apply to young adults in the context of FACT and Nordic countries should be explored. Following this, measurements can be developed, thus opening for evaluation studies of frameworks.

#### Education and training.

With social exclusion among young adults targeted as a major societal concern, the lack of education on social inclusion in health and social professions needs to be addressed. Basic education should provide fundamental knowledge on what social inclusion is and why it matters, both in terms of health promotion and preventive work, and as interventions. Training should be given on how it can be holistically addressed and what frameworks can be used to promote it. Knowledge of the promotion of social inclusion must be made available.

To improve staff understanding and promotion of social inclusion, targeted training could include workshops on the multidimensional nature of inclusion, emphasizing both objective factors like work and education and subjective factors related to psychological experiences and perceptions. Training should equip staff to address psychological and societal barriers. Through involving team staff, people with lived experience and other stakeholders, among these stakeholders who specialize in implementing and developing the FACT model, shared strategies could be developed. Continuous professional development modules focusing on best practices and innovative approaches would ensure staff remain updated and prepared to promote social inclusion systematically.

#### Policy makers, inclusive communities and context sensitive health and welfare services.

On a national level, and building on the reported lack of education on the subject and the concerns for social exclusion as stated in government policies, policies for higher education need to include knowledge of social inclusion as a prioritized competence for health and social professions. Following this, the guidelines for welfare and health services should include clear expectations and provide guidance as to how the services should work to promote social inclusion, going beyond interventions targeting specific domains. Building on research as called upon above, policies should more clearly integrate social inclusion as a fundamental component of mental health work, requiring service providers to adopt practices and frameworks to promote social inclusion. In addition, policies should highlight incentives and regulations, allowing services to work towards more context sensitive and inclusive practices.

Although much can be done on the individual level, there is a need to enhance the focus on building inclusive communities, e.g., through engaging stakeholders in the community [56,57]. Policy makers on a local level need to have financial resources and capacity to create inclusive practices. This includes fostering collaboration among diverse stakeholders such as public services, the private sector, non-profit organizations, and other community actors, to develop inclusive efforts. In addition, policies that promote intersectoral collaboration in a fragmented health and social care system are needed. Given previous research on FACT teams as bridging capacities between health and welfare services [53], studies should explore if FACT teams can form bridges to the general society, contribute to more inclusive communities and a more evenly distributed social capital.

### Strengths and limitations

A team approach was employed in the study with a combination of lived experience, psychological and sociological knowledge. The participants provided rich descriptions pointing to an acceptable degree of information power [58], however, it is possible that the study could have been strengthened by including a larger number of participants. The low number of participants may therefore have limitations on the transferability of the results. The participants worked in teams located in semi-urban areas, indicating that the findings might not apply whole cloth in other demographic contexts. This might constrain the generalizability of the findings. In addition, we did not explore team dynamics beyond interviewee comments, available resources, or institutional policies that could affect the FACT teams. This could have provided additional insights. Another limitation in the study could be including staff perspectives only. However, we have explored service users’ perspectives elsewhere [13,36]. Discussing these different perspectives in relation to each other should be explored in further research.
